# Targeting Transient Receptor Potential (TRP) Channels, Mas-Related G-Protein-Coupled Receptors (Mrgprs), and Protease-Activated Receptors (PARs) to Relieve Itch

**DOI:** 10.3390/ph16121707

**Published:** 2023-12-08

**Authors:** Merab G. Tsagareli, Taylor Follansbee, Mirela Iodi Carstens, Earl Carstens

**Affiliations:** 1 Laboratory of Pain and Analgesia, Ivane Beritashvili Center for Experimental Biomedicine, 0160 Tbilisi, Georgia; m.tsagareli@biomedicine.org.ge; 2 Department of Neuroscience, Johns Hopkins University, Baltimore, MD 21205, USA; tfollan1@jhmi.edu; 3 Department of Neurobiology, Physiology and Behavior, University of California, Davis, CA 95616, USA; mciodi@ucdavis.edu

**Keywords:** acute itch, atopic dermatitis, chronic itch, pruritus, psoriasis, scratching behavior

## Abstract

Itch (pruritus) is a sensation in the skin that provokes the desire to scratch. The sensation of itch is mediated through a subclass of primary afferent sensory neurons, termed pruriceptors, which express molecular receptors that are activated by itch-evoking ligands. Also expressed in pruriceptors are several types of Transient Receptor Potential (TRP) channels. TRP channels are a diverse class of cation channels that are responsive to various somatosensory stimuli like touch, pain, itch, and temperature. In pruriceptors, TRP channels can be activated through intracellular signaling cascades initiated by pruritogen receptors and underly neuronal activation. In this review, we discuss the role of TRP channels TRPA1, TRPV1, TRPV2, TRPV3, TRPV4, TRPM8, and TRPC3/4 in acute and chronic pruritus. Since these channels often mediate itch in association with pruritogen receptors, we also discuss Mas-related G-protein-coupled receptors (Mrgprs) and protease-activated receptors (PARs). Additionally, we cover the exciting therapeutic targets amongst the TRP family, as well as Mrgprs and PARs for the treatment of pruritus.

## 1. Introduction

Itch (or pruritus) is defined as an unpleasant sensation that provokes the desire to scratch, and is associated with an innate reaction to scratch away insects or plant spicules from the skin surface or to dig out invasive parasites. While everyday acute itch reflects an adaptive mechanism to maintain the integrity of the skin, chronic itch can adversely affect the quality of life to the point of suicidal ideation [1]. It is estimated that itchy skin conditions such as atopic dermatitis (AD) or psoriasis affect upwards of 10% or more of the general population, with associated annual health care and economic costs in the billions of dollars [2,3,4,5,6,7]. Chronic itch is thus a major health issue that demands more scientific attention. While major strides have been made in our understanding of itch mechanisms over the past few decades, the treatment of chronic itch remains challenging and requires the development of therapeutic approaches and pharmaceuticals targeting the currently known itch transducers and signaling pathways. 

Itch is generally classified as acute (<6 weeks) or chronic, lasting longer than six weeks. Chronic itch is a common symptom of multiple skin diseases, namely allergic contact dermatitis (ACD), AD, psoriasis, chronic urticaria, xerosis cutis, and other skin diseases such as prurigo nodularis, epidermolysis bullosa, lichen planus, actinic prurigo, morgellons disease, and aquagenic pruritus [8,9,10,11]. Chronic itch is divided into four categories: dermatological, systemic, neurological, and psychogenic [12]. Dermatological itch comes from skin conditions such as AD, psoriasis, and urticaria. Systemic itch can be caused by the pathology of other organs; for example, liver cholestasis and kidney dialysis. Neurological itch is induced by direct damage to the peripheral or central nervous system. Finally, psychogenic itch is associated with mental disorders [12,13]. 

Most types of chronic itch are resistant to antihistamines, so there is a pressing need to develop novel drugs other than antihistamines to treat itch. However, to date the management and treatment of itch remains challenging because therapeutic options have frequently been reported as inadequate [9,14,15,16]. 

In recent years, the role of various Transient Receptor Potential (TRP) channels and other receptors, including Mas-related G-protein-coupled receptors (Mrgprs) and Protease-Activated Receptors (PARs), have been identified as critical in transducing itchy stimuli into action potentials that are conducted over “pruiriceptive” primary afferent fibers into the nervous system. The aim of the present review is to briefly outline itch mechanisms with an emphasis on the role of TRP channels, Mrgprs and PARs in transducing pruritic stimuli, and especially to highlight the recent development of pharmaceuticals that act on these channels and receptors to potentially reduce itch.

## 2. The Transient Receptor Potential (TRP) Channels and Itch

**Figure 1:**
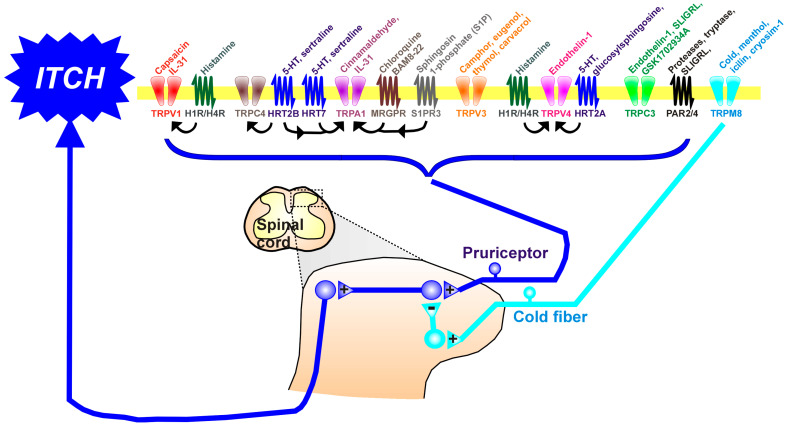
The upper part of the figure shows the variety of TRP channels and G-protein-coupled receptors (GPCRs), nearly all of which are expressed in the membranes of pruriceptive nerve endings in the skin. Many TRP channels and GPCRs are also expressed in skin keratinocytes. Above each TRP channel and GPCR are shown known ligands. Arrows indicate known interactions between GPCRs and TRP channels. The lower part of the figure shows afferent fibers of pruriceptors (blue) that enter the spinal cord via the dorsal roots, where they contact second-order neurons involved in processing itch. Itch signals project into ascending tracts (spinothalamic and spinoparabrachial) to reach higher centers involved in itch sensation. TRPM8-expressing cold fibers also enter the spinal cord to contact inhibitory interneurons (turquoise) that inhibit itch-transmitting spinal neurons. +: excitatory synapse; −: inhibitory synapse. Abbreviations: 5-HT: serotonin (5-hydroxytryptamine); HRT2A, 2B, 7: serotonin (5-hydroxytryptamine) receptor subtypes 2A, 2B, 7; IL-31: interleukin 31; Mrgpr: mas-related protein-coupled receptor; PAR: protease-activated receptor; S1PR3: sphingosine-1-phosphate receptor 3; TRP: transient receptor potential; TRPA1: TRP cation channel, subfamily A, member 1, TRPC3/4: cation channel, subfamily C (Canonical), Members 3 and 4, TRPM8: TRP Cation Channel Subfamily M (Melastatin) Member 8, TRPV1: TRP cation channel, subfamily V (Vanilloid), member 1; TRPV3: TRP cation channel, subfamily V (Vanilloid), member 3; TRPV4: TRP cation channel, subfamily V (Vanilloid), member 4.

TRP channels are involved in diverse sensory functions (smell, taste, touch, pain, temperature), including histamine-dependent and -independent itch. TRP superfamily ion channels, especially the TRP cation channel, subfamily A, member 1 (TRPA1), TRP cation channel, subfamily C (Canonical), Members 3 and 4 (TRPC3/4), TRP Cation Channel Subfamily M (Melastatin) Member 8 (TRPM8), TRP cation channel, subfamily V (Vanilloid), member 1 (TRPV1), TRP cation channel, subfamily V (Vanilloid), member 3 (TRPV3), and TRP cation channel, subfamily V (Vanilloid), member 4 (TRPV4), are key elements for signal transduction downstream of the G-protein-coupled receptors (GPCRs) and protease-activated receptors (PARs) (Figure 1). The opening of TRP channels allows calcium and sodium influx, leading to the depolarization of neuronal membranes and the opening of voltage-gated sodium channels to generate action potentials, thereby transmitting pruriceptive signals in primary afferents into the spinal cord to access ascending pathways to the brain to elicit itch sensations. 

Roles for TRP channels have been elucidated in complex diseases of the nervous, intestinal, renal, urogenital, respiratory, and cardiovascular systems in diverse functions including pain and itch, headache, pulmonary function, oncology, neurology, visceral organs, and genetic diseases [17]. Some TRP channels are involved in thermosensation and are stimulated by thermal stimuli across a specific temperature range [17,18]. 

Given the importance of TRPA1 and TRPV1 in mediating itch signaling, the investigation of these ion channels has been of considerable interest for their potential roles in contributing to chronic pruritis. Beyond the known expression of TRP channels in the nerve endings of primary afferent neurons, TRP channels have been found in keratinocytes, epidermis, and mast cells [19,20,21] and are upregulated in affected skin in several dermatological pathologies associated with chronic itch, including AD, psoriasis, and prurigo nodularis [21,22]. To determine the role of TRPA1 and TRPV1 in skin dysfunction, pharmacological and genetic knockout experiments have been performed in a variety of murine models of acute and chronic itch.

### 2.1. TRP Cation Channel, Subfamily A, Member 1 (TRPA1) in Acute Itch

TRPA1 is a non-selective cation channel for calcium ion influx, and widely expressed in the skin, sensory neurons, and many other tissues. TRPA1 is involved in sensory physiology and numerous systemic diseases. It is activated by a range of endogenous and exogenous stimuli, mainly including natural molecules such as allyl isothiocyanate (AITC, the main compound of mustard oil), eugenol, and cinnamaldehyde (CA). TRPA1 plays a significant role in mediating and regulating acute and chronic itching, and many itch-related GPCRs positively modulate TRPA1 [23]. Knockout mice (KO) lacking TRPA1 exhibited significantly reduced acute scratching elicited by chloroquine (CQ) and bovine adrenal medullary (BAM8-22) peptide [24] and sphingosine 1-phosphate (S1P) [25], as well as scratching in a model of chronic dry skin itch [26], indicating a role for TRPA1 in acute non-histaminergic as well as chronic itch. It was recently reported that TRPA1 inhibition reduced scratching behavior and calcium influx into dorsal root ganglion (DRG) cells elicited by a histamine H4 but not H1 receptor agonist, whereas TRPV1 inhibition reduced scratching and DRG neuronal calcium responses to both, suggesting that both TRPV1 and TRPA1 are involved in the transmission of histamine-induced itch [27,28]. In animal models, the study of DRG neurons has led to significant steps in our understanding of itch (and somatosensory) transduction. DRG neurons are a diverse population of primary afferent neurons which express different receptor mosaics allowing differentials in the responses to various modalities of stimulation (i.e., mechanical, warm, hot, cold, itch, etc.). The field has characterized several populations of itch-sensitive primary afferent neurons, allowing for a greater understanding of the neuronal circuitry as well as a deeper understanding of the intracellular signaling pathways relevant to itch transmission. While the DRG neurons have been well characterized in mice, human DRG neurons are less understood given the lack of available human DRGs for experimental usage. RNA expression studies have shown a significant correlation between the itch-relevant mouse and human DRGs, making the mouse a viable model organism [29]. 

We have recently reported that intraplantar injections of chloroquine, BAM8-22, and hexapeptide (Ser-Leu-Ile-Gly-Arg-Leu-_NH2_) (SLIGRL) elicited thermal hyperalgesia and mechanical allodynia in adult male mice [30]. Pretreatment with the TRPA1 antagonist (HC-030031) significantly reduced thermal hyperalgesia and mechanical allodynia elicited by chloroquine, BAM8-22, and SLIGRL, indicating that hypersensitivity effects developed by these non-histaminergic itch mediators require TRPA1 [10,30]. In another study, a moderate dose of formalin (1.25–5%) induced mixed wiping and scratching behavior in both mice and rats. However, the low dose of 0.3% formalin induced only scratch behavior, was histamine-independent, was significantly attenuated by a TRPA1 inhibitor (HC-030031), and was absent in TRPA1 KO mice [31]. 

In conclusion, TRPA1 is critical for acute non-histaminergic itch as well as chronic itch in dry skin, and is partially involved in acute histaminergic itch. TRPA1 represents a promising target for the development of antipruritics.

#### TRPA1 in Chronic Itch

AD is an inflammatory skin condition associated with intense itch, which generally develops in early childhood [32]. In AD, the occurrence of itch can precede the development of skin lesions and it is suggested to be neurogenic in origin [33,34]. In AD, thymic stromal lymphopoietin (TSLP) is released from epithelial cells and is critical to the atopic march triggering skin inflammation [35]. In the mouse, a genetic overexpression of TSLP in keratinocytes triggers itch and the development of AD-like skin. The injection of TSLP induced scratching behaviors, and KO of TRPA1 attenuated TSLP-evoked scratching, suggesting that TRPA1 is involved in TSLP-mediated itch in AD [36,37]. Topical application of the vitamin D analog calcipotriol (MC-903) produces AD-like pathology including increased itch and skin hyperplasia, and increased TSLP expression [38]. In TRPA1 KO mice treated with MC-903, there was a significantly reduced lesion area and fewer scratching bouts when compared to treated wildtype mice [39]. In AD, there is an increased expression of interleukin-13 (IL-13) which promotes inflammation and is associated with increased nerve ending innervation of lesioned skin [40]. In transgenic mice with an overexpression of IL-13, AD symptoms develop including increased itch, which is attenuated following the injection of the TRPA1 antagonist, HC-030031 [40]. In the DNCB (2,4-dinitro-chlorobenzene) model of AD, TRPA1 antagonism or KO resulted in a lower dermatitis score and fewer scratch bouts [41]. Glucosylsphingosine (GS) is an endogenous sphingolipid which is upregulated in the skin of AD patients. GS evokes the itch sensation and can activate neurons through a serotonin 2A receptor/TRPV4 interaction [42]. New evidence suggests that GS can activate neurons through a serotonin 2 receptor/TRPA1 but not TRPV1 interaction [43]. These results provide strong preclinical evidence for the importance of TRPA1 in the regulation of itch and skin inflammation in AD.

In the oxazolone and SADBE (squaric acid dibutyl ester) models of allergic contract dermatitis (ACD), the KO of TRPA1 reduced skin inflammation, skin edema, keratinocyte hyperplasia, and scratching behaviors [44,45]. Itch elicited by the application of the plant chemical urushiol, as commonly found in poison ivy, was reduced in TRPA1 but not TRPV1 knockout mice [44]. 

Psoriasis is an inflammatory skin disease which evokes an itch sensation in 60–90% of patients [46]. Psoriasis is mediated through the release of IL-17 from T helper-17 (Th17) cells, which induces feed forward inflammation [46,47]. In the mouse, a commonly used model for psoriasis is the topical application of imiquimod (IMQ), which induces many of the hallmarks of psoriasis [48]. Using the IMQ model in wildtype and TRPA1 KO mice, there was a significant reduction in the immune cells, inflammatory cytokines, skin inflammation and skin barrier defects in the TRPA1 KO mice [49]. Interestingly, when scratching behaviors were measured in the IMQ model, both male and female mice exhibited increased alloknesis (light touch-evoked scratching), but only male mice showed significantly increased spontaneous scratch bouts. In KO mice lacking TRPA1, spontaneous scratching behavior was not significantly affected while alloknesis scores were partially reduced in male mice [50]. Combined, these data imply that TRPA1 is necessary for the inflammation and disruption to the skin barrier in psoriasis and the development of alloknesis in males, but not for the underlying spontaneous scratching behaviors. Perhaps the inflammation of the skin in the IMQ model is mediated by TRPA1 expression via non-neuronal cells, while itch is regulated by endogenous itch mediators acting through several parallel pathways. 

A-967079, an analog of AP-18 that is an antagonist of TRPA1 [51], inhibited scratching behavior in mice induced by oxazolone and tacrolimus [44,52].

Despite the significant preclinical evidence showing the importance of TRPA1 in chronic itch, there are currently no clinical studies investigating TRPA1 antagonists for the treatment of AD (Table 1). There are a few studies which point to a role of TRPA1 in traditional medicines, with plant chemicals (Ke-Teng-zi; dictamnine) modulating the expression and sensitivity of TRPA1 and having positive outcomes for measurable AD symptoms including itch and skin lesions in mouse models [53,54]. 

### 2.2. TRP Cation Channel, Subfamily V (Vanilloid), Member 1 (TRPV1) in Acute Itch

Twenty-five years after its cloning [55], TRPV1 has become the first subfamily member linked to thermal pain and itch. Histamine activates primary sensory neurons via the histamine type 1 receptor (H1R) linked to TRPV1 [56] and histamine-evoked scratching is attenuated in knockout mice lacking TRPV1 [57].

We have recently found that an intraplantar injection of histamine in mice resulted in significant thermal hyperalgesia and mechanical allodynia ipsilaterally that persisted for 1 h. Pretreatment with the TRPV1 antagonist AMG-517, but not the TRPA1 antagonist HC-030031, significantly attenuated the magnitude and time course of thermal hyperalgesia and mechanical allodynia elicited by histamine, indicating that these effects are mediated by TRPV1 [10,30]. 

In addition, non-histaminergic pruritus such as that in cholestasis has also been related to TRPV1 sensitization by pruritogens [58]. TRPV1 also plays a role in non-histaminergic itch indirectly via PAR2 and PAR4, which are involved in chronic neurogenic inflammation. The latter sensitizes TRPV1 channels and induces itch [59]. 

#### TRPV1 in Chronic Itch

Understanding the influence of temperature fluctuations on skin diseases is critical to the development of measures for the prevention and treatment of allergic disorders. Nowadays, a number of studies have concluded that both cold and hot temperatures affect skin homeostasis and barrier function, and promote the development of AD. The temperature-driven activation of TRPV subfamily cation channels is involved in the induction of pruritus, flares, skin barrier dysfunction, the development of AD, and asthma attacks. The blocking of TRPV channels may attenuate temperature-mediated itch, skin barrier dysfunction, and the exacerbation of AD [60]. 

In the mice house dust mite (HDM) model of ACD, it was discovered that the administration of the TRPV1 antagonist, PAC-14028, reduced scratching and improved skin barrier function and recovery [61,62]. Similarly, in the SADBE model of ACD, the ablation of TRPV1-positive nerve fibers with the capsaicin analog, resiniferatoxin, or knockout of TRPV1 reduced scratching behaviors. SADBE directly activated HEK cells expressing TRPV1 (or TRPA1), inducing calcium transients. Interestingly, the antagonism or knockout of TRPV1 increased edema in the SADBE treatment [45]. These results suggest that TRPV1 promotes itch but negatively regulates inflammation in the SADBE model. 

Capsaicin, an agonist of TRPV1, has been used to treat the chronic itch of notalgia paresthetica [63,64], although the topical application of high-dose capsaicin induces discomfort and pain [65]. The topical application of TRPV1 antagonists, PAC-14028 and Asivatrep, resulted in a significant reduction in pruritus-related visual analog scale (VAS) scores in patients with AD [66,67,68]. Asivatrep (PAC-14028) was one of the first of a new class of non-vanilloid potent and selective TRPV1 antagonists. In a mouse AD model, oral treatment with asivatrep significantly reduced scratching behavior and suppressed the release of substance P (SP) through the inhibition of TRPV1 activation [61,62]. Asivatrep cream was well-tolerated and was not associated with clinically significant skin reactions and had an acceptable safety profile [69]. SB-705498 is another potent and selective TRPV1 antagonist [70], but had little effect on histamine- or cowhage-evoked itch in humans [71]. These results highlight the dissociation between itch and inflammation. While they often accompany each other in dermatological maladies, itch can occur without the presence of lesions and lesions can occur without itch. Nevertheless, TRPV1 remains a promising target for the development of antipruritic pharmaceuticals.

### 2.3. TRP Cation Channel, Subfamily V (Vanilloid), Member 2 (TRPV2)

TRPV2 is a nonspecific cation channel expressed in a subset of medium- to large-diameter DRG neurons. However, little information is available concerning its contribution to itch sensation and there is no concrete evidence yet whether TRPV2 is involved in various itch conditions [18]. Only one study reported that the *trpv2* gene was upregulated in the skin of patients with AD [22]. In a second study, the activation of TRPV2 in the human mast cell line (HMC-1) resulted in degranulation, a process through which endogenous pruritogens such as histamine are released [72]. 

### 2.4. TRP Cation Channel, Subfamily V (Vanilloid), Member 3 (TRPV3)

In the last few years, the TRPV3 channel has received much interest for its similarity to TRPV1. TRPV3 is a non-selective mainly calcium-permeable cation channel that is expressed in skin keratinocytes and is involved in multiple physiological and pathological functions of the skin, such as AD and Olmsted syndrome (OS). The human TRPV3 gene shows different degrees of sequence similar to TRPV1 and TRPV4. TRPV3 is expressed in diverse human tissues, among them the skin, DRG, spinal cord, brain, and testes [73]. 

Similar to TRPV1, the TRPV3 channel is a calcium-permeable, nonselective cation channel involved in itch and activated by temperature (>33 °C). Plant-derived camphor activates TRPV3 and sensitizes responses of the channel to warmth. Warm temperature stimulates TRPV3 in keratinocytes, releasing various inflammatory factors which activate pruriceptors in sensory neurons to transmit itch signals [18]. 

TRPV3 can also be activated by chemicals such as eugenol, thymol, and carvacrol—major components of oregano, savory, clove, and thyme. In mice, TRPV3 was not detected in DRG neurons, but it was detected in keratinocytes and is essential in causing allergic and pruritic dermatitis in rodents. In contrast, in primates, TRPV3 expression is also observed in DRG, TG sensory neurons, hypothalamus, and several nonneuronal tissues [74].

A recent study provided a clear link between TRPV3 and AD, showing that IL-31 induces B-type natriuretic peptide (BNP) synthesis and release from sensory neurons. BNP subsequently binds to natriuretic peptide receptor (NPR1) on keratinocytes to upregulate TRPV3 transcripts. This could increase the surface expression of TRPV3, resulting in heightened TRPV3 activity and increased serpin E1 release. In turn, serpin E1 activates sensory fibers in the skin and promotes itch transduction [75].

An increasing number of studies have shown that keratinocyte-expressed TRPV3 is involved in chronic pruritus and itch transmission [76]. Han et al. [77] reported that citrusinine-II, a plant-derived natural acridone alkaloid from *A. monophylla*, is a potent and selective antagonist of TRPV3 that has a strong antipruritic effect in both in vivo and in vitro experiments. These data indicate the potential of citrusinine-II-targeted therapy for itch [77].

In another study, it was found that keratinocytes isolated from patients with AD exhibited enhanced heat sensitivity and hyperactivity of TRPV3 [78]. TRPV3 was upregulated in the skin of the MC903-induced chronic AD mouse model. Heat stimulation to MC903-treated mice increased scratching behavior and produced higher levels of TLSP, nerve growth factor (NGF), prostaglandin E2 (PGE2), and IL-33 from the epidermis, which were attenuated by the pharmacologic inhibition of TRPV3 [78]. Finally, a patient with presumptive gain-of-function mutation of TRPV3 exhibited signs of OS, including chronic itch [79].

Overall, recent studies in rodents evaluated the relation of TRPV3 to itch in AD and psoriasis. However, less is known concerning the clinical relevance of TRPV3 in human studies. Further research is needed to reveal the roles of TRPV3 in human skin abnormalities in detail. 

### 2.5. TRP Cation Channel, Subfamily V (Vanilloid), Member 4 (TRPV4)

TRPV4 was originally described as an osmo- and mechanosensor [80]. An unexpected role for TRPV4 in itch came with the discovery that scratching behavior elicited by histamine [81,82] and serotonin [83] was reduced in KO mice lacking TRPV4, and that serotonin-evoked scratching and the activation of DRG neurons was reduced by a TRPV4 antagonist in wildtype mice [83] (for a review, see [84]).

Scratching elicited by the intradermal injection of endothelin-1 was also attenuated in TRPV4 KO mice [81]. Scratching elicited by glucosylsphingosine [42] and CA [85] was also reduced in TRPV4 KO mice. However, we did not observe any attenuation of scratching behavior elicited by the PAR-2 agonist SLIGRL in TRPV4 KO mice [83]. Signs of chronic itch in dry skin and contact dermatitis models were also reduced in KO mice lacking TRPV4 [84,86].

Recent studies suggest that chemicals from traditional Chinese medicines such as vitexin (apigenin-8-C-glucoside) [87] or cimifugin (from the herb *Saposhnikovia divaricata)* [88] may target TRPV4 to relieve acute and chronic itch. Thus, TRPV4 appears to be a good target for the development of drugs to alleviate itch.

### 2.6. TRP Cation Channel, Subfamily C (Canonical), Members 3,4 (TRPC3 and TRPC4)

TRPC3 is strongly expressed in DRG cells, and mice lacking TRPC3 exhibited significantly reduced scratching elicited by intradermal injections of endothelin-1, the PAR-2 agonist SLIGRL, and the TRPC3 agonist GSK1702934A [89], supporting a role for TRPC3 in non-histaminergic itch. The intradermal injection of the serotonergic antidepressant sertraline at a dose of 1 mmol was reported to elicit scratching behavior in mice via the 5HT-2B receptor and TRPC4 channel [90].

### 2.7. TRP Cation Channel, Subfamily M (Melastatin), Member 8 (TRPM8)

TRPM8 was originally described as a receptor for cold and menthol [91,92,93], conveying thermosensitivity in cold fibers. While skin cooling has been used for centuries to relieve itch, it was only recently shown that skin cooling and menthol can alleviate both histaminergic and nonhistaminergic itch-related behavior in mice in a TRPM8-dependent manner [94]. Cold fibers are thought to activate inhibitory spinal interneurons that suppress spinal itch transmission (Figure 1). Indeed, mice lacking a particular class of itch-inhibitory spinal interneuron (B5-I) exhibited a reduction in the antipruritic effect of menthol [95]. Recent clinical studies suggest that the cooling agent cryosim-1 may be antipruritic in various types of chronic itch, including scalp itch [96,97] (Table 1).

## 3. G-Protein-Coupled Receptors (GPCRs) and Itch Sensation

Pruriceptors express special protein molecules that can sense diverse itchy stimuli, including GPCRs which are predominantly expressed in the peripheral sensory nerve endings. They are members of the GPCR superfamily and are coupled to various G-proteins, through which they transduce their signals in second-messenger-mediated intracellular pathways [18,98]. 

At the beginning of this century, Dong and his group, using a cDNA subtractive screening approach, isolated a cDNA clone that is enriched in small-diameter DRG neurons and encodes a G protein-coupled receptor [99,100]. Because this receptor shares sequence homology with the proto-oncogene *Mas1*, they named this new gene *MrgA1* for “Mas1-related gene” which was later changed to MrgprA1 for “Mas1-related G protein-coupled receptor (Mrgpr)” [101]. 

Mrgprs are GPCRs encoded by the *Mrgpr* gene family and are key receptors involved in the regulation of itch (specifically, nonhistaminergic itch sensation), pain transmission, and inflammatory reactions. Cell bodies of sensory neurons encoding itch (pruriceptors) and pain (nociceptors) reside in DRG and TG. Most Mrgprs are associated with nociception and itch transmission, through their binding to various itch-inducing or pain-associated substances such as chloroquine, β–alanine, BAM8-22, or substance P (SP) [102,103].

**Figure 2:**
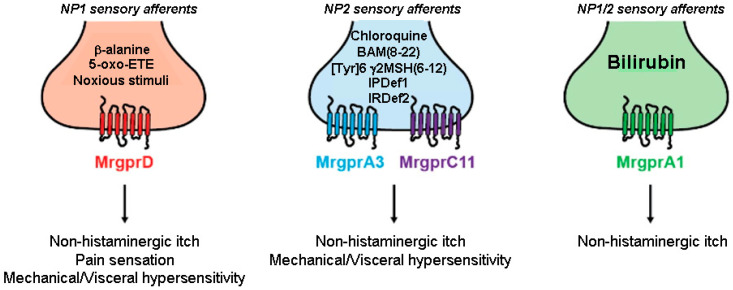
Small-diameter afferents neurons that express Mrgprs innervate the skin and can be divided into three distinct classes. MrgprD is mainly expressed by nociceptors belonging to the non-peptidergic family 1 (NP1) subpopulation (red) and is activated by β–alanine and 5-oxo-ETE causing non-histaminergic itch, mechanical and visceral hypersensitivity. MrgprA3 and MrgprC11 are Mrgprs expressed by pruriceptors belonging to the non-peptidergic family 2 (NP2, blue) and are activated by chloroquine and BAM8-22, and [Tyr]^6^-γ2-MSH(6-12), respectively. MrgprA1-positive sensory neurons (both NP1 and NP2 population) (green) are activated by bilirubin and are strongly linked to pruritus (adapted from [102]). Abbreviations: 5-oxo-ETE: 5-oxoeicosatetraenoic acid; BAM(8-22): Bovine Adrenal Medulla; IPDef1: IP defensin 1; IRDef2: IR defensin 2; Mrgpr: Mas-related G protein-coupled receptor, MSH: melanocyte-stimulating hormone; NP: non-peptidergic,: Tyr: tyrosine.

Figure 2 illustrates Mrgpr-expressing peripheral sensory neuronal terminals involved in pruriception and nociception. 

Nowadays, more than 50 members of the Mrgpr gene family have been identified in humans and rodents. Human receptors include six subfamilies, MRGPRX1–4 and MrgprD to G, whereas rodent receptors include eight subfamilies, MrgprA to H. The murine Mrgpr family is now known to comprise the MrgprA subfamily, with 14 members, the MrgprB and MrgprC families, together including 27 members, and the single-gene families, Mrgpr D, E, F, G, and H. MrgprA3 expressed in DRG neurons mediates chloroquine-induced itch sensation [104]. As for human receptors, MRGPRX1–4 are orthologous to the mouse MrgprA and B family members, while the other receptors (D to G) have identical murine orthologs. In particular, human MrgprX1 mediates the itch response to the pruritogens chloroquine and BAM8-22 that stimulate the murine MrgprA3 and MrgprC11, respectively [103,105].

Human MRGPRX2 is expressed exclusively by mast cells that are prominent in parts of the body such as the skin and which mediate allergic reactions. Mast cell activation and subsequent degranulation produce a significant inflammatory cascade, including the release of histamine [106,107,108] and the blockade of mast cells, and present a promising therapeutic target. Subcutaneous injections of MRGPRX2 agonists produce symptoms typical of mast cell activation, like weal, flare, and itch sensations, and all of these—including itch—are abolished by pretreatment with antihistamines. MRGPRX2 has a single mouse ortholog, called Mrgprb2, which is expressed exclusively by connective tissue mast cells, like MRGPRX2, and responds to multiple MRGPRX2 ligands. Mrgprb2 knockout mice lack mast cell responsiveness in vitro and in vivo to numerous dual MrgprX2/Mrgprb2 agonists, confirming their orthology [109]. The agonism of MrgprB2/X2 with PAMP1-20 (Pregnancy Associated Mouse Protein 1-20) results in the activation and degranulation of mast cells [109,110,111,112]. Furthermore, MRGPRX2 was elevated in the skin of atopic dermatitis and psoriasis patients [22] and in patients with chronic urticaria [113]. The injection of the MRGPRX2 agonist worsened skin reactivity in patients with chronic urticaria [114].

An MRGPRX2 antagonist (EP262) was developed by Escient Pharmaceuticals for the treatment of mast-cell-mediated diseases. Currently, this antagonist is in phase II trials for the treatment of AD and chronic urticaria. Evommune is also developing a MRGPRX2 antagonist for the treatment of chronic urticaria and “other indications”, but it has not yet reached phase I.

MGRPRX4 is expressed by human sensory neurons and is closely related to murine MrgprA1. Calcium recording experiments have shown that bile acids, which accumulate in cholestasis, activated both of these channels in a dose-dependent manner and produced itch behavior in humanized X4-expressing mice [110,111,112]. (Escient pharmaceuticals is developing an MRGPRX4 antagonist, currently in phase II, for the treatment of cholestatic/uremic itch.

Recent studies in HEK293T cell culture have revealed that a novel MRGPRX1 inhibitor, berbamine, potently inhibited chloroquine-mediated MRGPRX1 activation but did not alter the activity of other pruritogenic GPCRs. Furthermore, chloroquine-induced pruritus was significantly reduced by berbamine in a dose-dependent manner, but berbamine had no effect on itch in mice induced by histamine, PAR2–activating peptide, and deoxycholic acid [115].

**Figure 3:**
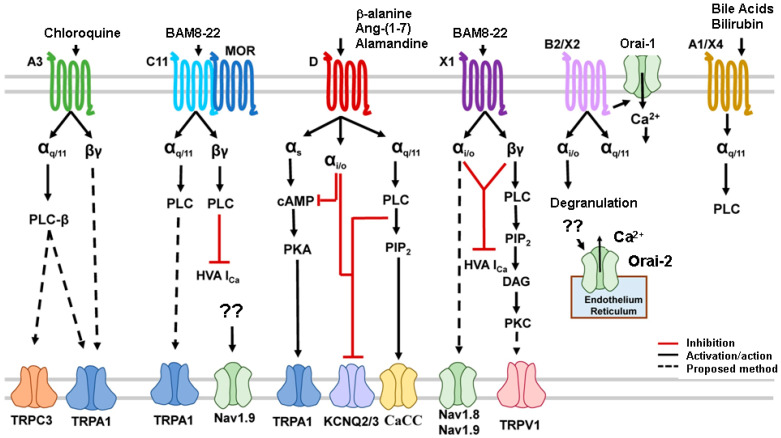
Mrgprs signaling pathways have been found to utilize Gαq/11, Gαi/o, Gαs, and Gβγ pathways. The activation of G proteins stimulates the intracellular signaling cascades and leads to the opening of transduction TRP, sodium, calcium, and potassium channels (adapted from [104]). Abbreviations: α_i/o:_ inhibitory G (guanine nucleotide-binding) protein α_Q/11_: stimulatory G (guanine nucleotide-binding) protein; α_s_: stimulatory G (guanine nucleotide-binding) protein; βγ: stimulatory G (guanine nucleotide-binding) protein; BAM: bovine adrenal medulla; CaCC: calcium-activated chloride channel; cAMP: cyclic adenosine monophosphate; DAG: diacyl glycerol; HVA: voltage activating channels; i_Ca_: calcium current; KCNQ: voltage-sensitive potassium channel; MOR: morphine; Nav: voltage-sensitive sodium channel; Orai: calcium release-activated channel protein; PKA: protein kinase A; PLC: phospholipase C; IP3: inositol 1,4,5 triphosphate; PIP2: phosphatidylinositol 4,5 biphosphate; TRPA1: TRP cation channel, subfamily A, member 1, TRPC3: cation channel, subfamily C (Canonical), Member 3; TRPV1: TRP cation channel, subfamily V (Vanilloid), member 1; ??: uncertain mechanism.

As stated above, some Mrgprs are involved in the mediation of itch, but scratching behavior induced by their activation is histamine-independent. Figure 3 illustrates Mrgpr signaling pathways and polymorphisms utilizing Gαq/11, Gαi/o, Gαs, and Gβγ pathways.

Overall, Mrgprs use a variety of endogenous ligands through diverse signaling pathways to encode and modulate various biological processes, including itch sensation, and Mrgpr polymorphisms may have a profound impact on the treatment of human skin diseases. Targeting Mrgprs appears to be a promising approach toward the development of novel antipruritics.

## 4. Protease Activated Receptors (PARs) and Itch Sensation

Protease-activated receptors (PARs) are G-protein-coupled receptors that are activated following the protease cleavage of part of the extracellular domain to expose a new amino terminus that acts as a ligand. Protease-activated receptor-2 (PAR-2) underlies the itch sensation evoked from protease-mediated pruritogens like mucunain in spicules of cowhage (*Mucuna pruriens*) [116,117]. While PARs have various physiological and pathophysiological functions in diverse organ systems, in the skin, PARs are involved in skin barrier homeostasis, inflammation, itch, and pain. PARs consist of four members, PAR1, PAR2, PAR3, and PAR4, and except for PAR3, regulate acute and chronic itch [11,118].

Various exogenous and endogenous proteases such as mucunain, trypsin, kallikreins (KLK), or tryptase have been demonstrated to be pruritogens in rodents and humans in vivo. Serine proteases like KLK, matriptase, prostasin, or tryptase have been implicated in various pruritic diseases, including AD, psoriasis, anaphylaxis-associated itch, dry skin itch, and renal insufficiency-associated itch [119]. It is thus important to understand the mechanisms by which PARs, in particular PAR2 and PAR4, regulate histamine-independent itch. Increased epidermal PAR2 activity is sufficient to drive many features of human AD in a mouse model [120]. Moreover, increased epidermal PAR2 activity facilitates skin sensitization upon exposure to pruritogens (histamine, SLIGRL, serotonin) and HDM extract. The remarkable effects of HDM were due to the direct activation of keratinocyte PAR2 by PAR2-activating proteases known to be present in HDM extracts and/or to other effects of HDM enabled and enhanced by the PAR2-driven skin barrier defect [120].

The cysteine protease cathepsin S from skin keratinocytes or resident/infiltrating immune cells acts as a pruritogen under chronic itch conditions. Intradermally injected human recombinant-cathepsin S or the PAR2 agonist, hexapeptide SLIGRL, behaved as pruritogens by causing scratching behavior in mice. Human recombinant-cathepsin S-induced scratching behavior was prevented by cathepsin S inhibitors and PAR2 antagonists and reduced by 50% in TRPV1 KO mice compared with wild-type mice, whilst no significant reduction in scratching behavior was observed in TRPA1 KO mice, concluding that cathepsin S acts as a pruritogen via PAR2 activation in TRPV1-expressing sensory neurons [121].

More recently, a PAR2 antagonist, the FSLLRY-_NH2_ peptide, was found to specifically activate MrgprC11 in a dose-dependent manner and also induce scratching behavior in mice. Moreover, FSLLRY moderately activated human MRGPRX1. In addition, FSLLRY stimulated downstream pathways including Gαq/11, phospholipase C (PLC), inositol triphosphate (IP3) receptor, and TRPC subfamily ion channels, increasing intracellular calcium levels [122].

A topical PAR2 inhibitor, methylbenzyl methylbenzimidazole piperidinyl methanone (MMP), was found to reduce cowhage-evoked itch in human subjects [123]. The antibiotic doxycycline was found to have an inhibitory action on PAR2 in keratinocytes [124]. Doxycycline reduced itch intensity in patients with pruritic acne [125]. In our search, we could not find any clinical trials involving PAR2 specific antagonists for the treatment of pruritis. While the studies mentioned above provide promise, more studies are needed to determine whether PAR-2 is an effective target for the development of pharmaceuticals to relieve itch.

In summary, a growing body of literature on the complex crosstalk between neuronal and immune cells that are involved in the development of acute and chronic itch has emphasized potential mediators and promising receptor therapeutic targets in the skin and peripheral nerve terminals comprised of GPCRs (Mrgprs and PAR2), interleukin (IL-31RA), TSLPR (TSLP receptor), histamine receptors (HR1, HR4), neurokinin (NK1R), tropomyosin receptor kinase (TrkA), and TRP channels (TRPA1, TRPC3/4, TRPV1-4) [126].

## 5. Conclusions

Emerging evidence clearly implicates TRP channels, Mrgprs, and PARs in a variety of itch-inducing mechanisms relevant to diseases which produce chronic itch. Since these channels and receptors are peripherally expressed and can mediate both inflammation and itch, they represent promising targets for the development of antipruritic pharmaceuticals. Only a few of the TRP channels discussed in this review have pharmaceutical agents (agonists and antagonists) currently in clinical trials. Several TRPV1 antagonists are in development for the treatment of pruritus, but with mixed results. Despite the strong preclinical evidence for TRPA1, there is a current lack of reports regarding pharmaceutical development for this promising target. More studies, including clinical trials of promising pharmaceutical agents that act at TRP channels, Mrgprs, and PARs, are sorely needed since most types of chronic itch are poorly treated by current therapeutics. Moreover, as we learn more about the central itch circuitry, it is hoped that pharmaceutical development will target other receptors, such as Gastrin Releasing Peptide (GRP)-expressing spinal neurons known to be involved in the central transmission of itch signals, in order to relieve itch. We hope that the current review sparks interest in pharmaceutical development to realize the untapped potential of targeting these receptors to relieve itch.

## Figures and Tables

**Table 1:** Pharmaceuticals targeting TRP channels, Mrgprs and PARs for the relief of various types of itch.

Itch Receptor	Agonists/ Activators	Antagonists/ Inhibitors	Disease Implications	Itch-Related Clinical Trial
TRPA1	AITC, eugenol, cinnamaldehyde	HC-030031, A967079	Non-histaminergic itch, Atopic dermatitis, allergic contact dermatitis, psoriasis	A967079-Completed
TRPV1	Capsaicin, resiniferatoxin	AMG-517, (Asivatrep) PAC-14028, SB705498	Histaminergic itch, atopic dermatitis, allergic contact dermatitis,	SB705498-Phase I, PAC-14028-Completed
TRPV2	NA	SKF96365	Atopic dermatitis	No ongoing trials
TRPV3	Camphor, eugenol	Citrusinine-II, KM001	Atopic dermatitis, Olmsted syndrome, Lichen simplex chronicus	KM-001-Phase I
TRPV4	NA	Vitexin, cimifugin	Serotonergic itch, dry skin itch, contact dermatitis	No ongoing trials
TRPC3	NA	NA	Non-histaminergic itch,	No ongoing trials
TRPC4	Sertraline	ML204, HC-070	Sertraline induced itch	No ongoing trials
TRPM8	Menthol, cryosim-1	Menthoxy-propanediol	Agonism functions as antipruritic, including scalp itch	No ongoing trials
MRGPRX2	SP, PACAP	EP262	Atopic dermatitis, chronic urticaria	EP262-Phase II
MRGPRX4	Bile acids, Bilirubin	EP547	Cholestatic itch, uremic pruritus	EP547-Phase II
MRGPRX1	BAM8-22	Berbamine	Chloroquine-induced itch	No ongoing trials
PAR2/4	Mucunain, tryptase, hexapeptide cathepsin S, FSLLRY-NH2	MMP, doxycycline	Atopic dermatitis, psoriasis, dry skin itch, contact dermatitis	Cowage-evoked pruritis, pruritic acne

## Data Availability

Data sharing is not applicable.

## Abbreviations

AD	atopic dermatitis
ACD	allergic contact dermatitis
AITC	allyl isothiocyanate
BAM8-22	bovine adrenal medulla peptide
BNP	B-type natriuretic peptide
CQ	chloroquine
CA	cinnamaldehyde
DRG	dorsal root ganglion
GS	glucosylsphingosine
GPCRs	G-protein-coupled receptors
HR1, HR4	histamine receptors
HDM	house dust mite
HMC	human mast cell (line)
ILs	interleukins
IMQ	imiquimod
IP3	inositol triphosphate
KLK	kallikreins
KO	knockout (mice)
MOR	morphine
Mrgpr	Mas-related G protein-coupled receptor
mROS	mitochondrial reactive oxygen species
NGF	nerve growth factor
NK1R	neurokinin receptor 1
NP	nonpeptidergic (receptors)
NPR1	natriuretic pepdide receptor
OS	Olmsted syndrome
PAR	protease-activated receptor
PIP2	phosphatidyl-inositol biphosphate
PLC	phospholipase C
PGE	prostaglandin E
SADBE	squaric acid dibutyl ester
SLIGRL	hexapeptide (Ser-Leu-Ile-Gly-Arg-Leu-_NH2_)
S1P	sphingosin 1-phosphate
S1PR3	S1P receptor 3
SP	substance P
TSLP	thymic stromal lymphopoietin
TRP	transient receptor potential (channels family)
TRPA	TRP ankyrin (subfamily)
TRPC	TRP canonical (subfamily)
TRPM	TRP melastatin (subfamily)
TRPV	TRP vanilloid (subfamily)
TrkA	tropomyosin receptor kinase A
TG	trigeminal ganglia
TNF	tumor necrosis factor
